# Comprehensive Evaluation of the Immune Response of Angus Cattle to Live Attenuated and Inactivated Goatpox Virus Vaccines

**DOI:** 10.3390/ani15172592

**Published:** 2025-09-03

**Authors:** Shijun Tian, Chao Chen, Lei Yang, Adili Abulaiti, Jinling Hua

**Affiliations:** 1 College of Animal Science, Anhui Science and Technology University, Chuzhou 239000, China; yjs2023440@ahstu.edu.cn (S.T.);; 2 Anhui Province Key Laboratory of Animal Nutritional Regulation and Health, Chuzhou 233100, China

**Keywords:** lumpy skin disease, live attenuated vaccine, inactivated vaccine, Angus cattle, gut microbiota

## Abstract

Lumpy skin disease (LSD) is a contagious animal disease caused by the *lumpy skin disease virus* (LSDV). LSD can be transmitted through direct, indirect and insect vectors, severely impacting global cattle production. To evaluate difference in immune response and gut microbiota of 30 healthy 16–18 months old Angus cattle, treated with live attenuated or inactivated goatpox vaccine virus strains. The cattle were randomly divided into three groups (10 animals per group): Group A—goatpox live attenuated vaccine; Group B—goatpox inactivated vaccine; Group C—control (saline). Blood samples were collected on days 14, 28, 42, and 56 post-vaccination to assess hematological parameters, serum biochemical indices, and antibody levels; rectal feces were collected on day 28 for *16S rRNA* analysis of gut microbiota. Results showed that, on day 28, both Group A and Group B reached their peak antibody levels (the log_10_ value of Group A was 2.6, and that of Group B was 2.7), with about 90% of the cattle in each vaccinated group testing antibody-positive. On day 42, Group B retained 90% seropositivity, whereas Group A declined to 80%. Significantly altered in vaccinated Groups (A and B) compared to controls on days 14 and 28 (*p* < 0.05). Hematological parameters (PLT, NEUT, and LYM) and serum biochemical indices (AST, TP, and GGT) were elevated early after vaccination but returned to baseline by days 42 and 56.Which returned to control levels by days 42 and 56. On day 28 post-vaccination, Vaccinated cattle showed significantly higher gut microbiome richness and diversity than unvaccinated controls (*p* < 0.05). At the phylum level, the dominance was observed in *Firmicutes* and *Bacteroidetes*: the relative abundances were 62.90% and 29.65% in Group A, 60.84% and 30.13% in Group B, and 49.99% and 39.73% in Group C, respectively. These findings indicate that the inactivated goatpox vaccine elicits a more durable and stable antibody response, maintaining higher specific antibody titers, and induces more pronounced shifts in the gut microbiota community structure at the phylum level compared with the live attenuated vaccine.

## Simple Summary

Lumpy skin disease (LSD), which is caused by the *lumpy skin disease virus* (LSDV), is a contagious bovine disease that has significantly impacted the global cattle industry. To elucidate the differential immune responses and gut microbiota profiles in cattle elicited by live and inactivated *capripoxvirus* strains, this study comparatively analyzed the integrated immune responses induced by live attenuated and inactivated goatpox vaccines. Alterations in the intestinal microbiota following vaccination were examined as a novel perspective to explore potential vaccine side effects or synergistic mechanisms. Additionally, antibody titers, complete blood counts, and serum biochemical parameters were concurrently assessed. The results demonstrated that, compared with the live attenuated vaccine, the inactivated vaccine elicited a more durable and stable antibody response, maintaining higher specific antibody titers. Furthermore, the inactivated vaccine induced more pronounced shifts in the gut microbial community structure at the phylum level. These findings provide a scientific basis for optimizing LSD immunization protocols in the cattle industry.

## 1. Introduction

Angus cattle (originating from Aberdeenshire and Angus in Scotland) come in two varieties, Black Angus and Red Angus. Owing to its excellent meat quality, wide adaptability, and relatively low breeding cost, Angus occupies a central position in the global beef industry [1]. Numerous studies also highlight the breed’s importance in genetic improvement programs [2,3]. With rising living standards and shifting consumer preferences, demand for high-quality beef (often associated with Angus beefs marbling, tenderness, and flavor) is increasing. Given Angus cattle’s high value, the industry must also prioritize disease prevention (e.g., against lumpy skin disease) to safeguard production. LSD is not only transmitted by direct contact with insect vectors, as well as through direct contact with infected animals or contaminated feed and water troughs [4].

Lumpy skin disease (LSD), also referred to as knopvelsiekte, is a contagious viral cattle disease caused by the *lumpy skin disease virus* (LSDV) [5]. It is an emerging transboundary animal disease capable of spreading beyond outbreak foci and becoming enzootic, thereby exerting significant economic impacts [6]. Lumpy skin disease (LSD) was first recorded in Zambia in 1929 and initially remained confined to African countries. By the 1940s, it had disseminated throughout southern Africa. In May 1988, Egypt reported its first outbreak, after which the disease progressively extended to the Middle East, then to Eastern Europe and Russia, and subsequently to the Balkans [7]. In 2019, novel cases were reported in South and East Asia, including Afghanistan, Pakistan, and India, where LSD was first detected in 2019 and has since become endemic [8]. Lumpy skin disease (LSD) is characterized by high morbidity and low mortality, with generalized nodular skin lesions. In males, testicular degeneration and scrotal edema can lead to temporary or permanent infertility. Pregnant cows may abort and may fail to resume normal estrous cycles for several months post-abortion [9]. Because of its rapid spread and potential for severe economic losses, the World Organization for Animal Health (WOAH/OIE) lists LSD as a notifiable disease. In 2022, China officially classified it as a Category II animal disease [10].

At present, live attenuated vaccines constitute the primary and effective means of preventing LSD [11]. These vaccines fall into two broad categories. The first comprises homologous attenuated vaccines based on LSDV strains, which are prepared directly from attenuated LSDV isolates and exhibit high specificity. Representative members include the South African Neethling strain vaccine and the Kenyan Sheep and Goat Pox virus strain vaccine (KSGP). Both of these vaccines have played pivotal roles in LSD control [12]. The second category consists of heterologous attenuated capripox vaccines derived from non-LSDV strains, mainly goatpox vaccines (e.g., Kedong, Gorgan, and AV41 strains) and sheeppox vaccines (e.g., Yugoslavian RM65, Romania, and Bakirköy strains) [13]. LSDV, *sheeppox virus* (SPPV), and *goatpox virus* (GTPV) exhibit strong serological cross-reactivity and share highly homologous antigenic components, enabling the induction of cross-protective immunity. Their physicochemical properties, morphological structures, and genomic architectures are also remarkably similar, thereby permitting the use of heterologous vaccines for LSD prevention [14]. However, prolonged use of attenuated live vaccines—whether the South African Neethling strain or attenuated goatpox and sheeppox strains—carries the risk that the vaccine virus may recombine with circulating field strains within the host, potentially restoring virulence or generating novel, more virulent variants. Sprygin [15] first documented recombination between two homologous attenuated vaccine strains (Neethling and KSGP). Bedeković [16] subsequently isolated LSDV from skin lesions, blood, and milk of animals vaccinated with attenuated vaccines. Consequently, attenuated live vaccines are not recommended for use in regions with low or no LSD risk.

This study aims to elucidate the differential immune responses and gut microbiota profiles of cattle vaccinated with live versus inactivated *goatpox virus* strains. Specifically, we monitored complete blood counts, serum biochemistry, and gut microbiota composition, integrating these data with specific humoral immunity (antibody titers) to deliver a comprehensive assessment of the immune response. By demonstrating how distinct vaccine preparations influence bovine immunity, the findings provide a scientific basis for optimizing LSD vaccination protocols in beef cattle operations.

## 2. Materials and Methods

### 2.1. Experimental Animals and Grouping Scheme

Thirty healthy 16–18 month old Angus cattle (mean body weight 387.5 ± 27.2 kg) from Xingya Farm (Qiaocheng District, Bozhou City, Anhui Province, China) were enrolled. Prior to post-vaccination, serum was collected from all animals and tested with a commercially available LSDV antibody (LSDV Ab) ELISA kit; all samples yielded negative results. They were randomly divided into three groups (10 animals per group): Group A—goatpox live attenuated vaccine; Group B—goatpox inactivated vaccine; Group C—control (saline). The detailed vaccination protocol is presented in Table 1.

### 2.2. Vaccines

The goatpox live attenuated vaccine (AV41 strain) was purchased from Harbin Pharmaceutical Group Biovaccine Co., Ltd. (Harbin, China). This product contains the *goatpox virus* strain (AV41 strain), which is diluted with physiological saline before injection. The virus content per dose for sheep is not less than 10^3.5^ TCID_50_. In the experiment, the dosage for immunizing cattle was equivalent to ten doses for sheep. The goatpox inactivated vaccine (AV41 strain) was purchased from China Animal Husbandry Industry Co., Ltd. (Beijing, China). This product contains the *goatpox virus* strain (AV41 strain), with a virus content of 10^6.2^ TCID_50_ per milliliter before inactivation. In the experiment, the dosage for immunizing cattle was 2 mL. The inactivation method for the goatpox vaccine is as follows: *Goatpox virus* AV41 strain was cultured using goat kidney suspension cells. After the cell culture reached a certain stage, the virus-containing culture was harvested. The harvested virus was then concentrated and purified to increase the content and purity of the viral antigen. The purified virus was mixed with the BEI inactivating agent and reacted for a certain period to inactivate the virus while retaining its antigenicity. The inactivated viral antigen was then mixed and emulsified with an adjuvant to produce the vaccine.

### 2.3. Antibody Level Sample Collection and Index Determination

On days 14, 28, 42 and 56 after post-vaccination, blood was collected from the tail root vein of all experimental animals and blood was collected into 10 mL vessels, incubated at ambient temperature for 30–60 min, centrifuged at 3000 rpm for 10 min, and the serum stored at −20 °C. The antibody was determined using a *lumpy skin disease virus* antibody (LSDV Ab) ELISA kit (Cat. No. ANG-E101142LB, Lot No. 2025021039B) purchased from Nanjing Aoqing Biotechnology Co., Ltd. (Nanjing, China). ELISA antibody detection was performed according to the manufacturer’s instructions, and the results were interpreted. The negative control OD value was less than 0.2, the positive control OD value was greater than 0.8, the positive judgment (cut-off value): the negative control OD value + 0.25, the sample OD value was greater than the threshold, judged as positive, otherwise negative.

### 2.4. Blood Index Sample Collection and Index Determination

On days 14, 28, 42 and 56 after post-vaccination, blood was collected from the caudal vein of all experimental animals and placed in a 10 mL EDTA anticoagulant tube. Routine blood indices, such as red blood cell count (RBC), platelet count (PLT), white blood cell count (WBC), neutrophil ratio (NEUT), and lymphocyte ratio (LYM), were measured using a Mindray automatic blood analyzer (Model No. BC-5180CRP, Shenzhen Mindray Biomedical Electronics Co., Ltd., Shenzhen, China). A blood biochemical analyzer (Model No. BECKMAN COULTER AU680, Beckman Coulter, Inc., Brea, CA, USA) was utilized to assess serum levels of aspartate aminotransferase (AST), total protein (TP), blood urea nitrogen (BUN), uric acid (UA), gamma-glutamyl transferase (GGT), and creatinine (CREA), and other biochemical indicators.

### 2.5. Gut Microbiota Sample Collection and Index Determination

On day 28 post-vaccination, the feces of all experimental animals were collected, placed in a 5 mL freezing tube, and then placed in an incubator with dry ice. Following total DNA extraction from the sample, primers were designed based on the conserved region, and a sequencing adapter was appended to the primer’s end. The V3-V4 region of the bacterial *16S rRNA* gene was amplified using PCR. The primer information was F: 5′-ACTCCTACGGGAGGCAGCA-3′ and R: 5′-GGACTACHVGGGTWTCTAAT-3′. Amplicons were purified, quantified, and normalized to generate sequencing libraries. Libraries passing quality control were sequenced on an Illumina NovaSeq 6000 platform (Illumina, Inc., San Diego, CA, USA). The high-quality sequences were subjected to clustering or denoising to delineate operational taxonomic units (OTUs) or amplicon sequence variants (ASVs), hereinafter referred to as “features.” Species-level classification was then assigned based on the sequence composition of each feature. Based on the feature analysis results, the samples were classified at each taxonomic level. Finally, alpha diversity analysis was used to study the species diversity within individual samples. The Ace, Chao1, Shannon and PD _ whole _ tree indexes of each sample were counted, and the alpha diversity index of the sample was evaluated using QIIME2 2020.6 [17] software.

### 2.6. Data Statistical Analysis

The experimental data were collated in Excel 2016 and analyzed with SPSS 27.0 statistical software. One-way analysis of variance (ANOVA) was conducted, followed by multiple comparisons using Duncan’s method. Results are presented as mean ± standard error of the mean (SEM). Correlation analysis was conducted using Spearman’s correlation coefficient. Data visualization was performed using Origin 2024 software. Statistical significance was established at *p* < 0.05, whereas *p* > 0.05 denoted no significant difference.

## 3. Results

### 3.1. Results of Antibody Level Determination

**Figure 1:**
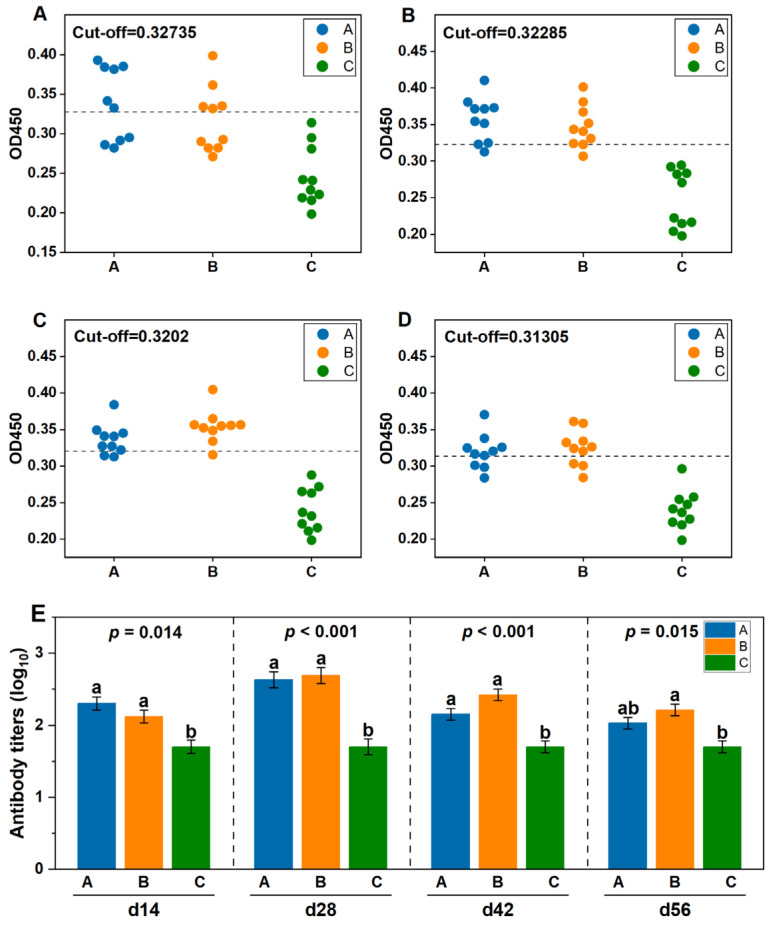
Antibody levels were detected after vaccination: (**A**) The antibody OD value on day 14; (**B**) The antibody OD value on day28; (**C**) The antibody OD value on day 42; (**D**) The antibody OD value on day 56; (**E**) Antibody levels after vaccination. Greater than the cut-off value is positive; peer data shoulder the same letter or no letter said the difference was not significant (*p* > 0.05), different letters denote significant differences (*p* < 0.05).

On days 14, 28, 42 and 56 after post-vaccination, serum antibody levels (log_10_ value) were detected using ELISA. As shown in Figure 1. The antibody levels in Group C were negative. Compared with Group C, the antibody levels of Groups A and B were significantly increased on day 14 (*p* < 0.05), with positive rates of 60% and 50%, and antibody levels of 2.3 and 2.12, respectively. On day 28, the antibody levels in Groups A and B were significantly increased (*p* < 0.05), with both groups showing a 90% positive rate, and the antibody levels were 2.63 and 2.69, respectively. On day 42, the antibody levels of Groups A and B were significantly increased (*p* < 0.05), the positive rates were 80% and 90%, and the antibody levels were 2.15 and 2.42, respectively. On day 56, the antibody levels in Group B were significantly increased (*p* < 0.05). The positive rates for Group A and Group B were both 70%., respectively, and the antibody levels were 2.03 and 2.21, respectively. From the whole period of the experiment, there was no significant difference in antibody levels between Group A and Group B on days 14, 28, 42, and 56 *(p* > 0.05).

### 3.2. Blood Routine Test Results

The effect of the vaccine on blood routine after post-vaccination of Angus cattle is shown in Table 2. The results showed no significant difference in routine blood indices between the groups on day 56 (*p* > 0.05). Compared with Group C, PLT, NEUT, and LYM in Groups A and B were significantly increased on days 14 and 28 (*p* < 0.05). On day 42, the PLT and LYM levels in Group B increased significantly (*p* < 0.05). Additionally, on day 14, the WBC count in Group A was significantly higher than in Groups B and C (*p* < 0.05); on day 28, the WBC count in Group B was significantly higher than in Group C, but did not differ significantly from that in Group A (*p* < 0.05). Throughout the experimental period, the blood indexes showed significant differences between groups on days 14 and 28, including PLT, NEUT and LYM.

### 3.3. Serum Biochemical Determination Results

The effect of the vaccine on serum biochemical after post-vaccination of Angus cattle is shown in Table 3. On day 56, no significant differences in serum biochemical indices, including CREA levels, were observed among the groups (*p* > 0.05). Compared with Group C, the levels of AST, BUN, UA, and GGT in Groups A and B were significantly increased on day 14 (*p* < 0.05). On day 28, AST and TP levels in Groups A and B were significantly increased (*p* < 0.05). On day 42, TP in Group B was significantly increased (*p* < 0.05). Throughout the experimental period, the serum biochemical indices showed the most significant differences between the groups on days 14 and 28,including AST, TP, and GGT.

### 3.4. Gut Microbiota Venn Diagram Analysis

**Figure 2:**
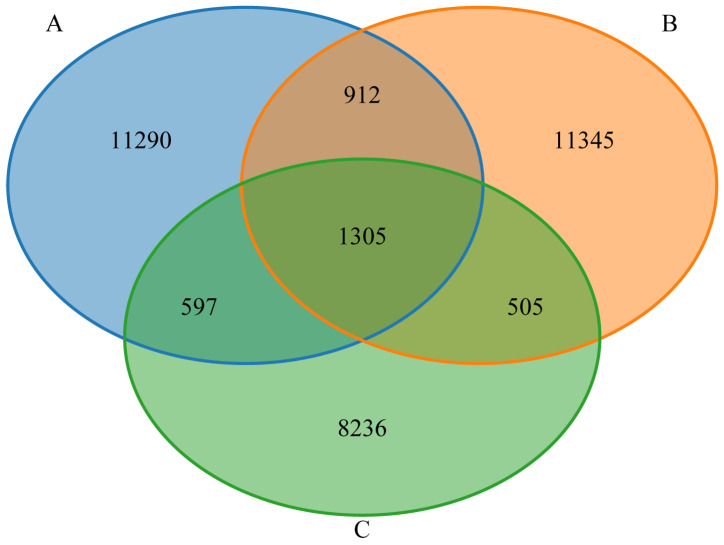
Feature Venn graph. The colors of distinct ellipses signify disparate groups, the overlapping segment denotes the count of shared features among groups, and the non-overlapping segment corresponds to the count of unique features per group.

As shown in Figure 2, the gut microbiota of Groups A, B, and C shared 1305 features. Groups A and B shared 2217 features, Groups A and C shared 1902 features, and Groups B and C shared 1810 features. In addition, Group A possessed 11,290 unique features, Group B 11,345, and Group C 8236. Overall, the gut microbiota of Groups A and B exhibited greater species richness than that of Group C, whereas the species richness between Groups A and B is similar.

### 3.5. Alpha Diversity Analysis of the Gut Microbiota

**Figure 3:**
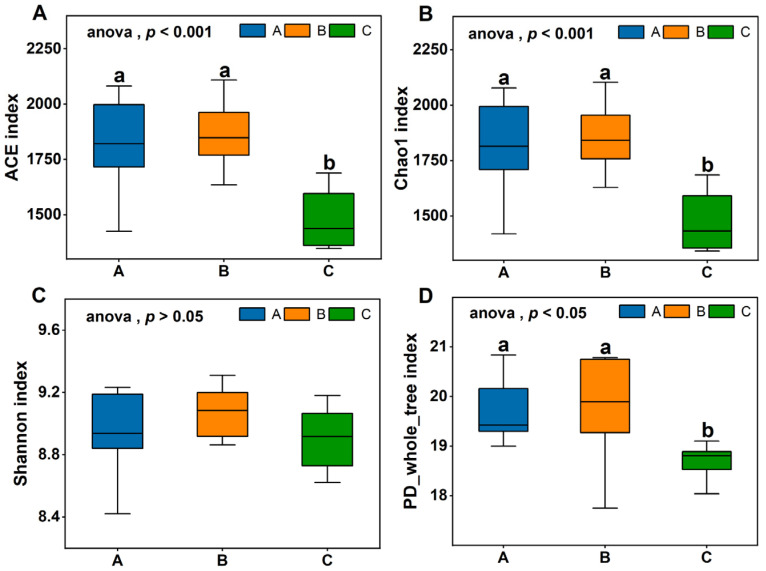
Alpha diversity index difference box plot between groups. (**A**) ACE index; (**B**) Chao 1 index; (**C**) Shannon index; (**D**) PD_whole_tree index. Peer data shoulder the same letter or no letter said the difference was not significant (*p* > 0.05), different letters denote significant differences (*p* < 0.05).

The Ace and Chao 1 indices are commonly used to estimate community richness; higher values indicate greater species representation. The Shannon index is employed to assess species diversity, with larger index values corresponding to higher species diversity [18]. The PD_whole_tree index is a measure of phylogenetic diversity calculated based on the phylogenetic tree. Higher values of this index indicate greater community diversity. In this study, the coverage of each sample group exceeded 0.9996, which attests to the reliability of the test results. As shown in Figure 3. The Ace index, Chao1 index, and the PD_whole_tree index was significantly higher in Groups A and B compared to Group C (*p* < 0.05), with no significant difference between Groups A and B (*p* > 0.05). Similarly, the Shannon index was higher in Groups A and B than in Group C, although the difference was not statistically significant (*p* > 0.05), these findings suggest that Groups A and B exhibited comparable species richness and diversity in their gut microbiota, both significantly exceeding those in Group C.

### 3.6. Species Richness at the Phylum Level

**Figure 4:**
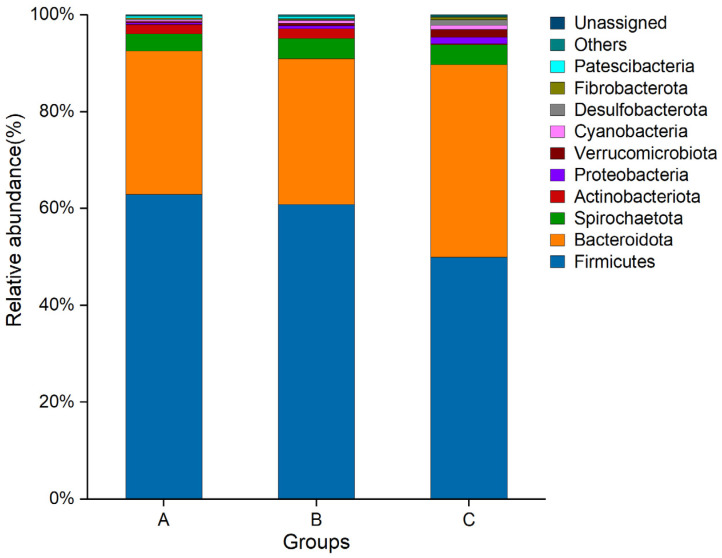
Species richness at the phylum level. Each color signifies a unique species, and the length of the associated bar reflects the relative abundance of that species.

As shown in Figure 4. The gut microbiota in Groups A, B, and C were dominated by *Firmicutes* and *Bacteroidetes*. The relative abundances of Group A were 62.90% and 29.65%, respectively. The relative abundances of Group B were 60.84% and 30.13%, respectively. The relative abundance of Group C was 49.99% and 39.73%, respectively.

### 3.7. Correlation Analysis Results

**Figure 5:**
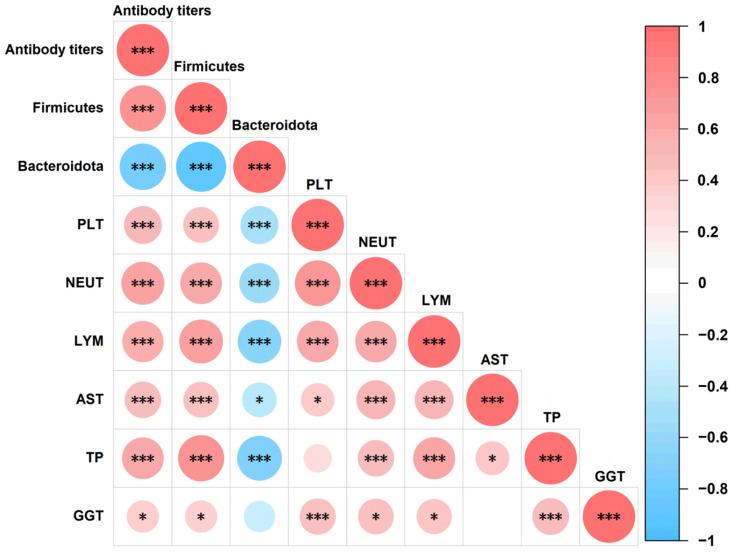
Correlation analysis of antibody level, *Firmicutes*, *Bacteroidota*, PLT, NEUT, LYM, AST, TP and GGT. The red means positive correlation, and the bule means negative correlation. * *p* < 0.05, *** *p* < 0.001.

On day 28, Spearman correlation analysis was conducted to assess relationships between antibody levels and the following parameters: *Firmicutes, Bacteroidota*, PLT, NEUT, LYM, AST, TP, and GGT. As shown in Figure 5. Antibody levels were positively associated with *Firmicutes*, PLT, NEUT, LYM, AST, TP, and GGT (*p* < 0.05) and negatively associated with *Bacteroidota* (*p* < 0.05). Additionally, PLT, NEUT, LYM, AST, TP, and GGT levels exhibited positive correlations with *Firmicutes* (*p* < 0.05) and negative correlations with *Bacteroidota* (*p* < 0.05).

## 4. Discussion

This study compared the effects of live attenuated and inactivated goatpox vaccines on antibody titers, complete blood counts, serum biochemistry, and gut microbiota in Angus cattle. The findings delineate the differential immune responses and microbiota alterations elicited by live attenuated versus inactivated *goatpox virus* strains, providing a comprehensive assessment of the immune responsiveness of Angus cattle to both vaccine formulations.

Throughout the entire study period, antibody titers in Groups A and B were significantly higher than those in Group C (*p* < 0.05), indicating that both live attenuated and inactivated *goatpox virus* strains can elicit a durable antibody response. The antibody level in Group A was higher than that in Group B on day 14, which showed that the antibody response was produced earlier and the antibody level was high. The antibody levels reached their highest value on day 28, and then gradually decreased on days 42 and 56, which is consistent with the characteristics of the live attenuated vaccine to quickly activate the immune system, similar to other international studies [19,20]. Simultaneously, we found that not all immunized cattle could detect neutralizing antibody responses. The antibody positivity rate was 60% on day 14 and 90% on day 28. It has been reported that the seroconversion rate of serum antibodies after vaccination of cattle with attenuated vaccines such as goatpox and sheeppox is 20–60% [21]. The results of our study are high, which may be attributed to the breed and immune dose of the cattle. To effectively prevent and control the *lumpy skin disease virus*, most countries generally administer five times the dose of the sheeppox attenuated vaccine for subcutaneous injection in live cattle [22,23]. Saudi Arabia, which uses the Romanian SP.Among vaccine, needs to vaccinate cattle every 6 months to enhance the immune effect, and with an inoculation dose that is 10 times that of sheep [24]. According to the current vaccination protocol of the farm, this study used 10 times the dose of goatpox live attenuated vaccine to immunize Angus cattle, and also achieved a good immune effect.

The inactivated goatpox vaccine employed in this study used the AV41 strain as the antigen and was produced via an advanced suspension-culture technology, representing the same viral strain administered to Group A. The results showed that The antibody level in Group B was lower than that in Group A on day 14, reached the highest value on day 28, and the antibody level remained high on day 42. At the same time, the decrease was more gradual than that of Group A on days 42 and 56, indicating that the antibody response elicited by the inactivated goatpox vaccine was comparable to that of the goatpox live attenuated vaccine. We found that the inactivated goatpox vaccine maintained a high antibody conversion rate after booster immunization. The antibody conversion rate was 50% on day 14, and the antibody conversion rate it was 90% on days 28 and 42. The antibody level in Group A decreased to 77% of the peak on day 56, which is consistent with the long-term maintenance of memory cells dependent on live attenuated vaccines [25]. The antibody level in Group B still maintained 82% of the peak on day 56, suggesting that the adjuvant slowed down the decay rate by enhancing the antibody affinity [26,27]. The antibody titer of Group C was always ≤1:50, which was significantly different from that of the vaccine group (*p* < 0.05). Subsequent research should further investigate the long-term antibody persistence of the two vaccines and more comprehensively compare and analyze the immune response.

On day 14, Group A exhibited significantly different routine blood and serum biochemical indices compared to Group B (*p* < 0.05). By day 28, Group B demonstrated greater changes in these indices than Group A (*p* < 0.05), and the attenuation was more gradual on days 42 and 56, which was similar to the change pattern of antibody level. This may be because the live attenuated vaccine simulated infection through the rapid activation of lymphocyte reactions to achieve early high antibody production, but the attenuation was faster [28]. Group B compensated for the deficiency of adaptive immune activation through high-dose antigen and adjuvant administration and achieving a more gradual antibody decay curve [29]. On days 14 and 42, Group B exhibited significantly higher PLT levels compared to the other groups (*p* < 0.05), and the highest value observed on day 28. This phenomenon may be due to the fact that platelets secrete molecules, such as CD40L, which directly promote B cell differentiation and antibody production [30]. The increase in PLT levels was synchronized with the change in antibody level in Group B. On day 28, NEUT in Group B increased to 88.09%, which was significantly greater than Groups A and C (*p* < 0.05), suggesting that adjuvants may overactivate neutrophils to release neutrophil extracellular traps (NETs), leading to the risk of tissue damage [31]. Adjuvants in inactivated vaccines are essential for amplifying vaccine-induced immune responses by modulating the magnitude and characteristics of both humoral and cellular immunity, resulting in a significant prolongation of antibody secretion [32]. This process was accompanied by a continuous increase in TP, which was consistent with the finding that TP in Group B maintained a high level on days 28 and 42 in this study. The changes in all indicators recovered on day 56, confirming the vaccine’s safety. Varduhi Hakobyan [33] found in the study of the serological response of cattle immunized with the attenuated sheeppox vaccine that no swelling at the injection site or any other side effects of the vaccine were observed after vaccination, and the body temperature of all cattle did not increase and did not deviate from physiological indicators. From an industry perspective, some blood cell counts and enzymes increased early after vaccination, but quickly returned to baseline levels, reducing concerns about repeated vaccination of high-value beef cattle.

The intestinal tract contains a large number of microorganisms. Recent studies have shown that intestinal microorganisms have an important impact on the life activities of the host and contribute to digestion and absorption, stress resistance, and immune response of the host [34,35]. Intestinal microbial communities maintain host health by participating in material metabolism, nutrient absorption, immune response, and disease defense [36]. Under normal physiological conditions, gut microbiota can regulate the host immune response through specific components, such as lipopolysaccharides, lipoproteins, and metabolites, and promote the development of the immune system [37]. Acetic, propionic, and butyric acids are the most abundant short-chain fatty acids (SCFAs) in mammals. *Bacteroidetes* and *Firmicutes* dominate as the most prevalent phyla in the intestines. *Bacteroidetes* members predominantly produce acetate and propionate, while *Firmicutes* primarily generate butyrate in the intestine [38]. The abundance of *Bacteroidetes* and *Firmicutes* in the intestine is related to the specific immunity elicited by vaccines. Therefore, it is speculated that the short-chain fatty acids produced by such intestinal microorganisms act on the host immune system and affect this process. Studies have shown that specific microbial groups influence vaccine efficacy. These microorganisms are positively correlated with the host immune response and the abundance of immune cells, whereas *Bacteroidetes* in the intestine and Pseudomonadales belonging to *Proteobacteria* show the opposite correlation [39,40]. These findings are consistent with the results of the current study. The correlation heat map indicates that the immunization of Angus cattle with the goatpox live attenuated vaccine (Group A) and the inactivated goatpox vaccine (Group B) resulted in elevated *Firmicutes* abundance and reduced *Bacteroidetes* abundance in the gut microbiota. Antibody titers exhibited significant positive correlations with *Firmicutes*, PLT, NEUT, LYM, AST, TP, and GGT, and a significant negative correlation with *Bacteroidetes*, these correlations suggest that the gut microbiota enhances systemic immunity through microbe–host interactions. Butyrate derived from *Firmicutes* has been shown to potentiate T follicular helper (Tfh) cells and sustain germinal-center reactions [41]. Live attenuated vaccines undergoes transient replication, and the resulting acute inflammatory milieu may—via mechanisms yet to be elucidated—transiently reshape the microbial community, leading to fluctuations in the *Firmicutes*/*Bacteroidetes* ratio. In contrast, the adjuvanted inactivated vaccine elicits a mild, Th2-biased inflammation that mitigates intestinal hypoxia and preserves barrier integrity [42]. This allows *Firmicutes* to change only modestly, so the F/B ratio does not increase significantly. Because inflammation remains mild, intestinal barrier integrity is better preserved, resulting in more efficient butyrate uptake. Consequently, even a modest abundance of *Firmicutes* is sufficient to activate the hepatic *FXR*/*PPAR-γ* pathway, driving adequate TP production and antibody maturation [43]. We therefore hypothesize that vaccination alters the bovine gut microbiota, which in turn modulates vaccine immunogenicity and consequently shifts antibody titers. The booster dose of the inactivated vaccine, advantaged by its adjuvant, further enhances antibody affinity—constituting the central mechanism underlying its superior immunogenicity.

Using *16S rRNA* sequencing, this study provides an initial exploration of the differential gut microbiota profiles in cattle vaccinated with live attenuated versus inactivated *capripoxvirus* strains. Future work should employ metagenomics or transcriptomics to delineate the underlying immune pathways and to establish causal links between specific microbial metabolites and vaccine efficacy.

## 5. Conclusions

The present study confirms that both live attenuated and inactivated *goatpox virus* vaccines elicit robust antibody responses. Compared with the live attenuated vaccine, the inactivated formulation exhibits a more gradual antibody decline and induces more pronounced shifts in the dominant gut bacterial phyla. Moreover, neither vaccine provoked overt inflammatory reactions nor adversely affected hepatic or renal function. These findings indicate that the inactivated goatpox vaccine is a viable immunization strategy for the clinical prevention of lumpy skin disease, providing an important reference for optimizing LSDV vaccination protocols and evaluating immunization efficacy in cattle.

## Figures and Tables

**Table 1:** Vaccination cattle test program.

Groups	Numbers	Inoculation Dose ^1^	Second Immune	Inoculation Time
A	10	2 mL	none	a.m.
B	10	2 mL	14 d	a.m.
C	10	2 mL	none	a.m.

^1^ Group A: goatpox live attenuated vaccine virus content per milliliter was 5 × 10^3.5^ TCID_50_ AV41 strain; Group B: the virus content per milliliter of goatpox vaccine before inactivation was 10^6.2^ TCID_50_ AV41 strain; Group C: saline injection; TCID_50_: half of the tissue culture infection dose.

**Table 2:** Angus cattle blood routine index determination results.

Items ^1^	Groups			SEM ^2^	*p*-Value
	A	B	C		
Day 14					
RBC (10^12^/L)	6.84 ^b^	7.27 ^ab^	7.63 ^a^	0.12	0.005
PLT (10^9^/L)	216.31 ^b^	250.97 ^a^	156.63 ^c^	8.70	<0.001
WBC (10^9^/L)	7.15 ^a^	6.49 ^b^	6.23 ^b^	0.13	0.004
NEUT (%)	76.53 ^a^	70.82 ^b^	68.51 ^c^	0.75	<0.001
LYM (%)	16.11 ^a^	14.90 ^a^	12.23 ^b^	0.44	<0.001
Day 28					
RBC (10^12^/L)	7.49	7.53	7.58	0.15	0.973
PLT (10^9^/L)	202.00 ^b^	270.90 ^a^	160.94 ^c^	9.65	<0.001
WBC (10^9^/L)	6.69 ^ab^	7.10 ^a^	6.13 ^b^	0.16	0.049
NEUT (%)	75.80 ^b^	88.09 ^a^	69.24 ^c^	1.78	<0.001
LYM (%)	18.60 ^a^	19.20 ^a^	11.54 ^b^	0.76	<0.001
Day 42					
RBC (10^12^/L)	7.54	7.74	7.66	0.25	0.951
PLT (10^9^/L)	161.50 ^b^	184.73 ^a^	162.13 ^b^	3.94	0.017
WBC (10^9^/L)	6.83	6.91	6.17	0.16	0.107
NEUT (%)	70.73	71.64	69.91	0.64	0.555
LYM (%)	12.50 ^ab^	13.53 ^a^	11.28 ^b^	0.32	0.009
Day 56					
RBC (10^12^/L)	7.46	7.54	7.77	0.18	0.781
PLT (10^9^/L)	164.08	168.65	163.48	4.52	0.888
WBC (10^9^/L)	6.42	6.44	6.35	0.18	0.976
NEUT (%)	69.41	70.81	69.34	0.60	0.541
LYM (%)	12.20	12.70	11.51	0.42	0.529

^a–c^ Antibody levels after vaccination: peer data shoulder the same letter or no letter said the difference was not significant (*p* > 0.05), different letters denote significant differences (*p* < 0.05). ^1^ Red blood cell count (RBC), Platelet count (PLT), White blood cell count (WBC), Neutrophil ratio (NEUT), Lymphocyte ratio (LYM). ^2^ Standard error of the mean.

**Table 3:** Determination results of serum biochemical indexes of Angus cattle.

Items ^1^	Groups			SEM ^2^	*p*-Value
	A	B	C		
Day 14					
AST (U/L)	105.28 ^a^	79.26 ^b^	60.03 ^c^	3.58	<0.001
TP (g/L)	69.80 ^a^	59.36 ^b^	57.06 ^b^	1.16	<0.001
BUN (mmol/L)	5.92 ^a^	5.34 ^b^	2.55 ^c^	0.29	<0.001
UA (umol/L)	98.49 ^a^	78.05 ^b^	49.67 ^c^	4.06	<0.001
GGT (U/L)	54.26 ^a^	32.63 ^b^	23.07 ^c^	2.46	<0.001
CREA (umol/L)	122.46	118.70	109.78	2.34	0.071
Day 28					
AST (U/L)	72.77 ^a^	68.04 ^a^	60.69 ^b^	1.50	0.002
TP (g/L)	80.04 ^a^	74.49 ^b^	57.31 ^c^	1.83	<0.001
BUN (mmol/L)	3.94	4.33	2.56	0.33	0.061
UA (umol/L)	72.89	68.80	50.14	4.70	0.106
GGT (U/L)	32.94 ^ab^	41.15 ^a^	23.06 ^b^	2.50	0.008
CREA (umol/L)	116.26	114.36	110.22	1.62	0.309
Day 42					
AST (U/L)	63.62	65.76	60.55	1.05	0.121
TP (g/L)	68.75 ^ab^	77.75 ^a^	57.20 ^b^	2.86	0.008
BUN (mmol/L)	3.21	3.69	2.55	0.24	0.160
UA (umol/L)	56.80	60.32	49.41	2.18	0.111
GGT (U/L)	30.48	31.34	23.16	1.80	0.124
CREA (umol/L)	110.79	112.68	110.88	1.06	0.726
Day 56					
AST (U/L)	62.18	63.32	60.77	0.87	0.504
TP (g/L)	60.91	65.34	57.07	2.32	0.358
BUN (mmol/L)	2.54	3.01	2.44	0.17	0.374
UA (umol/L)	51.61	56.54	50.11	1.46	0.174
GGT (U/L)	24.77	26.79	23.84	1.15	0.580
CREA (umol/L)	111.25	113.08	110.06	1.34	0.667

^a–c^ Antibody levels after vaccination: peer data shoulder the same letter or no letter said the difference was not significant (*p* > 0.05), different letters denote significant differences (*p* < 0.05). ^1^ Aminotransferase (AST), Total protein (TP), Blood urea nitrogen (BUN), Uric acid (UA), Gamma-glutamyl transferase (GGT), Creatinine (CREA). ^2^ Standard error of the mean.

## Data Availability

The raw data of bacterial 16S rRNA sequencing obtained from the intestinal feces of Angus beef cattle has been submitted to the NCBI Sequence Read Archive (SRA) database (Accession number: PRJNA1273273).
